# The diagnosis of urinary tract infections in young children (DUTY): protocol for a diagnostic and prospective observational study to derive and validate a clinical algorithm for the diagnosis of UTI in children presenting to primary care with an acute illness

**DOI:** 10.1186/1471-2334-12-158

**Published:** 2012-07-19

**Authors:** Harriet Downing, Emma Thomas-Jones, Micaela Gal, Cherry-Ann Waldron, Jonathan Sterne, William Hollingworth, Kerenza Hood, Brendan Delaney, Paul Little, Robin Howe, Mandy Wootton, Alastair Macgowan, Christopher C Butler, Alastair D Hay

**Affiliations:** 1 Academic Unit of Primary Health Care, School of Social and Community Medicine, University of Bristol, Canynge Hall, 39 Whatley Road, Clifton, Bristol, BS8 2PS, UK; 2 South East Wales Trials Unit (SEWTU), Institute for Translation, Innovation, Methodologies and Engagement, School of Medicine, Cardiff University, 7th Floor Neuadd Meirionnydd, Heath Park, Cardiff, CF14 4XN, UK; 3 Wales School of Primary Care Research (WSPCR), Institute of Primary Care & Public Health, School of Medicine, Cardiff University, 5th Floor Neuadd Meirionnydd, Heath Park, Cardiff, CF14 4XN, UK; 4 School of Social and Community Medicine, University of Bristol, Canynge Hall, 39 Whatley Road, Clifton, Bristol, BS8 2PS, UK; 5 Department of Primary Care and Public Health Sciences, King’s College London, School of Medicine, 5th Floor Capital House, 42 Weston Street, London, SE1 3QD, UK; 6 Department of Primary Medical Care, University of Southampton, Aldermoor Close, Southampton, SO16 5ST, UK; 7 Specialist Antimicrobial Chemotherapy Unit, Public Health Wales Microbiology Cardiff, University Hospital Wales, Heath Park, Cardiff, CF14 4XW, UK; 8 North Bristol NHS Trust, Southmead Hospital, Westbury-on-Trym, Bristol, BS10 5NB, UK

**Keywords:** Urinary Tract Infection, Children, Primary care, Point-of-care-test, Dipstick test, Near-patient testing, Diagnosis, Economic models

## Abstract

**Background:**

Urinary tract infection (UTI) is common in children, and may cause serious illness and recurrent symptoms. However, obtaining a urine sample from young children in primary care is challenging and not feasible for large numbers. Evidence regarding the predictive value of symptoms, signs and urinalysis for UTI in young children is urgently needed to help primary care clinicians better identify children who should be investigated for UTI. This paper describes the protocol for the Diagnosis of Urinary Tract infection in Young children (DUTY) study. The overall study aim is to derive and validate a cost-effective clinical algorithm for the diagnosis of UTI in children presenting to primary care acutely unwell.

**Methods/design:**

DUTY is a multicentre, diagnostic and prospective observational study aiming to recruit at least 7,000 children aged before their fifth birthday, being assessed in primary care for any acute, non-traumatic, illness of ≤ 28 days duration. Urine samples will be obtained from eligible consented children, and data collected on medical history and presenting symptoms and signs. Urine samples will be dipstick tested in general practice and sent for microbiological analysis. All children with culture positive urines and a random sample of children with urine culture results in other, non-positive categories will be followed up to record symptom duration and healthcare resource use. A diagnostic algorithm will be constructed and validated and an economic evaluation conducted.

The primary outcome will be a validated diagnostic algorithm using a reference standard of a pure/predominant growth of at least >10^3^, but usually >10^5^ CFU/mL of one, but no more than two uropathogens.

We will use logistic regression to identify the clinical predictors (i.e. demographic, medical history, presenting signs and symptoms and urine dipstick analysis results) most strongly associated with a positive urine culture result. We will then use economic evaluation to compare the cost effectiveness of the candidate prediction rules.

**Discussion:**

This study will provide novel, clinically important information on the diagnostic features of childhood UTI and the cost effectiveness of a validated prediction rule, to help primary care clinicians improve the efficiency of their diagnostic strategy for UTI in young children.

## Background

Acute illness in young children is one of the commonest reasons for seeking health care worldwide. Reported rates of urinary tract infection (UTI) in children consulting for any acute condition vary widely (from 2% to 20% depending on setting and inclusion criteria) and most of this research has been hospital based [1,2]. Only one study has systematically sampled urine from sequentially presenting acutely unwell children in primary care, and found UTI prevalence of 4% [3]. However, that study was not adequately powered to estimate the predictive value of symptoms and signs with adequate precision.

UTI may be missed in as many as 50% of young children presenting to primary care [4,5]. The clinical diagnosis of UTI in young children is difficult because: (1) pre-verbal (predominantly under 3 years) children cannot articulate symptoms and present with the same non-specific symptoms (e.g. fever, irritability, vomiting and poor feeding) when suffering from a wide range of illnesses [6]; (2) identifying dysuria and changes in urination frequency in children wearing nappies (diapers) is difficult; (3) obtaining urine samples is often challenging and time consuming for parents[1] and costly to the health service [7,8]; and (4) the National Institute for Health and Clinical Excellence (NICE) does not recommend routine urine dipstick testing in children under 3 years because of a lack of research evidence as to its diagnostic value [9]. UTI diagnosis is therefore often delayed [6], missed [4] or symptoms attributed to other causes (such as otitis media) [10].

UTIs in young children may cause acute morbidity and recurrent symptoms that may indicate functional and anatomical abnormalities. In some young children, UTI may lead to renal scarring [11], leading to poor renal growth, recurrent pyelonephritis, impaired glomerular function, early hypertension, end stage renal disease [12] and pre-eclampsia [13-15]. Some experts, therefore, recommend aggressive, early antibiotic treatment for symptoms suggestive of UTI in young children to prevent renal scarring [16].

Dramatic reductions during the second half of the 1990s and early 2000s in antibiotic prescribing for children with upper respiratory infections [17] may have reduced serendipitous treatment of undiagnosed UTI and the consequent prevention of renal scarring. However, antibiotic prescribing in the UK may now be on the increase [18]. NICE promotes early recognition and treatment to prevent short-term suffering and possibly serious long-term complications [9]. However, increased urine sampling will increase costs, consultation length and frequency of consultations in primary care. Clinicians will therefore only increase their sampling rates if evidence shows this really does improve the identification of UTI among the many acutely unwell children consulting primary care.

To date there is a lack of evidence as to the clinical predictors of UTI in young children. A meta-analysis [1] including 8,837 mostly pre-verbal children from 12 studies, showed that fever, non black race, a history of a previous UTI, temperature higher than 40°C, and suprapubic tenderness were the findings most useful for identifying those with a UTI. Uncircumcised boys were also more likely to have a UTI. While individual symptoms and signs were helpful in the diagnosis of a UTI, they were not sufficiently accurate to definitely rule it in, however a combination of findings could identify infants with a low probability of UTI [1]. The applicability of this review to UK general practice is limited because: (1) included studies were set in the US private and emergency care system where consultation and investigation threshold differs from UK primary care and other health care systems that are free at the point of delivery; (2) children had to either already have symptoms of UTI or fever ≥38°C, so many subtle symptoms and signs may not have been considered; (3) urine sampling was by catheter or suprapubic aspiration (which is not conducted in most primary care settings world-wide and from which any bacterial growth is regarded as significant); (4) diagnostic criteria used were different (≥10^4^ colony forming units per millilitre (CFU/mL)) to UK practice (≥10^5^ CFU/mL); (5) the relationship between ethnicity and UTI could be confounded; and (6) none of the studies included in the meta-analysis checked the external validity [19] of the findings, meaning that estimates of association could be inflated [20].

A more recent review of primary care based paediatric studies using urine culture as the reference standard found that no individual symptom or sign, or any combination of symptoms or signs, was sufficient to rule in a diagnosis of UTI, although some (e.g. increased capillary refill time, no fluid intake, and supra-pubic tenderness) appear to warrant urine testing and empirical treatment while awaiting culture confirmation [2]. Furthermore, a number of symptoms and signs did not appear to have diagnostic value, including some of those included in the NICE guidelines (e.g. poor feeding and vomiting). Some symptoms, signs and proposed clinical prediction rules were associated with a sufficiently low UTI probability to rule out UTI, thereby removing the need to obtain a urine sample [1-3].

We found only one clinical algorithm derived from primary research for the diagnosis of UTI in young children. The research included febrile girls aged under two years in one US Emergency Department [21] and was validated in a case–control study in a different Emergency Department [22]. They found that more than three findings of: aged less than 12 months; white race; temperature of ≥39°C; absence of any other likely source of fever; or fever for 2 or more days gave an area under the curve of 0.72, a sensitivity of 88% (95% CI: 79% to 94%) with a false-positive rate of 70% (95% CI: 61% to 79%).

An additional issue for the diagnosis of UTI in children is uncertainty as to the best criteria for microbiological diagnosis of UTI in this age group. Historically this has been based on a colony count of bacteria in the urine with a cut-off of ≥10^5^ colony forming units (cfu)/mL of a uropathogen indicating infection. However this was derived from studies in adult women [23] and its applicability to children has been questioned by NICE [9] and others. Current guidance for microbiological diagnosis of UTI in children is at variance. The UK National Standard Method suggests that colony counts of ≥10^3^ cfu/mL of a single species may be diagnostic of UTI in voided urine and that a pure growth of between 104–105 cfu/mL is indicative of UTI in a carefully taken specimen [24]. For midstream specimens, the European Association of Urology, suggests a cut-off of ≥10^4^ cfu/mL if associated with symptoms, but ≥10^5^ cfu/mL if symptoms are absent; lower cut-offs are suggested for PSA and bladder catheterisation samples [25]. However guidance from the American Academy of Pediatrics suggests that both urinalysis suggesting infection (pyuria and/or bacteriuria) plus the presence of ≥ 5x10^4^cfu/mL of a uropathogen are required for a diagnosis of UTI, although these guidelines are for urine specimens obtained through catheterization or an SPA, which would not be routine in the UK [26].

### Additional value of dipstick testing in young children

A 2006 Health Technology Assessment (HTA) review found there was inadequate evidence on the diagnostic performance of dipstick tests for protein or blood for children aged under 5 years old. The combination of a positive test for both nitrite and leucocyte esterase (LE) was most accurate for ruling in UTI (pooled LR + 28.2 (95% CI: 17.3 to 46.0)), and a negative test for both nitrite and LE was most accurate for ruling out UTI (pooled LR- 0.20 (95% CI: 0.16 to 0.26)) [27]. The NICE UTI guideline development group concluded that there was insufficient evidence to recommend the use of dipstick urine tests for children under 3 years [9].

### Economic impact of UTI

UTI is the fourth most common reason overall for prescribing antibiotics in UK general practice, accounting for approximately 8% of all antibacterial prescriptions . However, UTI is currently infrequently diagnosed in children [28]. Whilst the unit costs of laboratory testing and antibiotic prescribing are relatively low [27], the *economic implications* of new clinical algorithms for urine sampling and testing may be substantial in young children because of: (1) the large numbers of children who present with non-specific symptoms who might be candidates for urine sampling and testing; (2) the cost, to healthcare services and to patients, of subsequent diagnostic tests (e.g. ultrasound, Micturating Cystourethrogram (MCUG) and Dimercaptosuccinic Acid (DMSA) scans) used to further evaluate children with recurrent/atypical UTI [9]; (3) the substantial societal costs and utility detriments of a missed diagnosis that leads to rare but serious complications of UTI; and (4) the wider, long-term population impact of diagnostic algorithms on antibiotic prescribing and resistance [29].

The few economic evaluations of methods for diagnosing UTI in young children [27,30] have primarily evaluated ‘which tests to use?’ rather than ‘who to test?’ The 2006 HTA review [27] evaluated 79 permutations of dipstick, cultures, ultrasound, and MCUG, and identified four testing strategies most likely to be cost-effective, although the optimal strategy differed by gender and age group. Current NICE guidance on testing strategies for UTI in children under 3 years is not based on evidence of cost-effectiveness [9].

### Research objectives

In summary, rigorous evidence regarding the predictive value of symptoms, signs and urinalysis for UTI in young children is urgently needed to help primary care clinicians better identify UTI. Furthermore, since obtaining urine samples is especially challenging in children aged before their fifth birthday, the resulting algorithm will be constructed to answer two separate questions: first, which children warrant urine sampling? And second, can point of care dipstick urinalysis help clinicians determine which samples should be sent for laboratory culture? The algorithm will then be the subject of a validation study. Furthermore, since changes in the frequency with which urine samples are requested has implications for parents and for healthcare services, analyses will model the economic impact (from the NHS and societal perspectives) of GP judgement versus diagnostic algorithm guided diagnosis and management with respect to the cost per correctly identified UTI, cost per symptomatic day avoided and the cost per quality adjusted life year.

The Diagnosis of Urinary Tract Infection in Young Children (DUTY) study protocol has the following research objectives:

1. To develop candidate clinical prediction rules that accurately identify children presenting in primary care with an acute illness in whom a urine sample should be obtained, based on socio-demographic factors, medical history, symptoms and signs.

2. To assess whether dipstick urinalysis for nitrite, leukocyte esterase, protein, blood and glucose gives additional diagnostic information to objective (1) in the identification of urine samples that should be sent for laboratory analysis.

3. To model cost-effectiveness from NHS and societal perspectives of the candidate clinical prediction rules.

4. To compare contamination rates for different urine sampling methods.

## Methods/Design

### Ethical and governance approval

Multi-centre approval was granted by the South West Southmead Research Ethics Committee (previously Southmead Research Ethics Committee, then South West 4 REC), Ref #09/H0102/64. Research and Development (R&D) approval has been granted for all sites taking part in the study.

### Design

DUTY is a 3-year, multicentre, diagnostic accuracy study to derive and validate a cost effective algorithm for the diagnosis of UTI in children under 5 presenting to primary care with an acute illness.

Children are eligible if they are aged before their fifth birthday and present to primary care with a new acute illness episode of less than or equal to 28 days duration. A Case Report Form (CRF) will be completed for all eligible, consented children and a urine sample obtained. The prevalence of UTI will be determined on laboratory culture. An algorithm will be derived and validated in separate samples of children.

### Setting

This UK study will be implemented from four research centres at the Universities of Bristol, Cardiff, Southampton and King’s College London. Each centre will recruit children from primary care, defined as any NHS facility providing first-point-of-contact face-to-face advice for parents of unwell children (GP practices, Walk-in-Centres (WIC), and Children’s Emergency Departments (CED)).

### Study procedures

#### Primary care sites/GP practice recruitment

Primary care sites will be recruited by each study centre covering both urban and rural areas across England and Wales. Two models of recruitment will be offered: (1) Option 1, in which the majority of the recruitment procedures will be undertaken by a dedicated Research Nurse (RN) or Clinical Studies Officer (CSO) external to the site; and (2) Option 2, in which recruitment will be undertaken entirely ‘in house’ by the primary care site’s clinical team. From now on, members of staff taking informed consent for the DUTY study will be referred to as “recruiting clinicians”. Dedicated RNs/CSOs providing external support for sites will be referred to as “DUTY recruiters”, while members of practice staff involved in option 2 recruitment will be referred to as “site-based recruiters”.

### Recruiting staff

The study grant will provide full-time equivalent DUTY recruiter posts across all four study centres, which will be supplemented by additional DUTY recruiter posts provided by local Primary Care Research Networks (PCRNs) and Comprehensive Local Research Networks (CLRNs) (in England) and by the National Institute for Social Care and Health Research – Coordinating Research Centre (NISCHR-CRC) (in Wales). These DUTY recruiters will be available to provide Option 1 support to primary care sites, and to support autonomously recruiting Option 2 sites through the provision of expert training, mentoring and problem-solving.

### NHS microbiology laboratory recruitment

The participation of any primary care site in recruitment to the study will depend on the support and participation of the local NHS microbiology laboratory to which the site routinely sends urine samples. In each area of recruitment, the local NHS laboratory will be approached and service level agreements put in place prior to involvement in the study.

### Participant recruitment

The recruitment process is summarised in 1.

**Figure 1:**
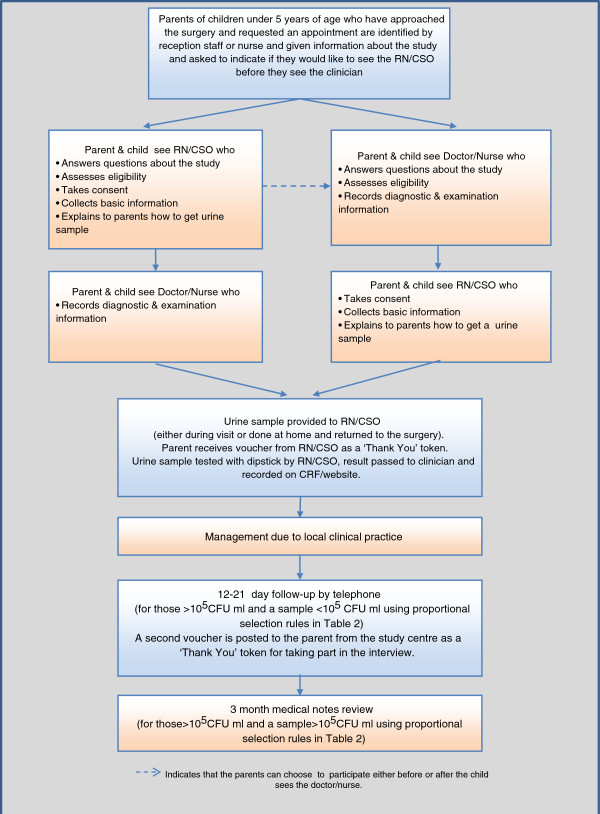
DUTY participant flow diagram.

### Registration and consent

Parents and children may be invited to take part in the study in a number of ways:

1. Where possible, primary care sites will mention the study to parents of children under 5 when they phone for an appointment, and ask them to come to the surgery 15 minutes early to receive further information.

2. Where the study was not raised at the time of making the appointment, parents of children already booked in may be phoned and told about the study and invited to attend a little earlier.

3. If they cannot be contacted by telephone, they will be approached on arrival at the site, given information sheets and asked if they would be happy to see a recruiting clinician to discuss participation.

Once the parent has indicated they are happy to discuss the study, the recruiting clinician will explain study participation answer any questions that the parent may have, ensure that they fully understand the implications of participation, and check the child’s eligibility. If the child is eligible and the parent agrees to participate, written informed consent will be obtained from the parent. If the parent wishes to see the GP before consenting, the recruiting clinician will arrange for this to happen, with the parent and child returning to the recruiting clinician afterwards to complete recruitment. Where possible the recruiting clinician will recruit the participant while they are waiting to see the GP, in order that the parent and child are not delayed. However, if more practical or convenient, the recruiting clinician may offer, with the parent’s permission, to visit the family later the same day at their home to complete recruitment. If the parent is not interested in hearing more about the study, no further approach will be made.

### Non-registration

A screening log of all children aged before their fifth birthday, who are attending for care and whose parents are approached by the recruiting clinician to invite participation in the study, will be compiled. Details will be recorded as to their eligibility, whether consent was given or declined, and reason for declining to participate.

### Participant eligibility

Table 1 details the inclusion and exclusion criteria for the study. Since ruling out UTI may be as important as ruling it in, the study inclusion criteria were designed to be as broad as possible. Therefore, children consulting with other ‘obvious’ causes for their symptoms such as otitis media or bronchiolitis, as well as those with a history of previous UTI and known abnormalities of the urinary tract, learning difficulties, or re-consulting for an existing illness are all included as long as none of the exclusion criteria apply.

**Table 1:** DUTY eligibility criteria

**Children will be included if:**	**Children will be excluded if:**
Aged before their fifth birthday.	Aged 5 years and above.
Presenting at a participating NHS primary care site.	Parents are unable or unwilling to assist with study.
Presenting with an acute (≤28 days) illness as the main reason for the parent to have requested an appointment.	Illness longer than 28 days duration.
	Presenting with trauma as a predominant concern.
Presenting with at least one ’constitutional’ symptom or sign identified by NICE [9] as a potential marker for UTI – that is, fever, vomiting, lethargy/malaise, irritability, poor feeding and failure to thrive *and/or* at least one urinary symptom identified by NICE [9] as a potential marker of UTI – that is, abdominal pain, jaundice (children <3 months only), haematuria, offensive urine, cloudy urine, loin tenderness, frequency, apparent pain on passing urine and changes to continence.	No urinary or constitutional symptoms as defined by NICE [9] and listed in the left hand column.
	Known neurogenic (e.g. spina bifida) or surgically reconstructed bladder or urinary permanent or intermittent catheterisation (for whom different bacterial concentration cut points are used).
	Taking any antibiotics in the last 7 days.
	Taking immunosuppressant medication (e.g. anti-rejection drugs, oral or intramuscular steroids or chemotherapy).
	Already recruited into the DUTY study.
	Involved in current research or have recently (within 28 days) been involved in any research prior to recruitment.
	There will be no recruitment to the study after the last NHS laboratory transport of the day has departed from that primary care site on Fridays.
	For recruitment at A&E settings only: children will not be eligible if their presentation at A&E is a direct result of GP referral.

The study will include parents who speak other (non-English) languages. Parent information sheets and consent forms will be translated into other languages as required by participating GP practices (e.g. Welsh, Polish and Brazilian Portuguese). For languages less commonly spoken in the UK, particularly for those in which oral translation is more useful than written translation (e.g. Somali), translational services will be accessed, where possible, via interpreters employed by recruiting primary care sites to support patient-clinician communications. Where these services are not available, translational services will be provided via Language Line.

### Collecting urine samples and dipstick testing

The recruiting clinician will attempt to obtain a urine sample from the children of consenting parents during the recruitment visit. If this is not possible, the recruiting clinician will ask the parent to collect the sample at home, give them the appropriate equipment and explain how to collect it. To minimise contamination, urine samples will be obtained using the ‘clean catch’ where this is possible. Where this is not possible, the ‘nappy pad’ method (which involves cleaning the child’s perineum and inserting a sterile pad into the nappy to soak up urine, for a maximum period of 1 hour) will be used, as described by Liaw et al. [31] and as recommended by the recent NICE guidelines [9]. Urine sampling can be underway whilst the recruiting clinician completes the study CRF.

The recruiting clinician will retrieve the urine sample, test it with a urine dipstick (Siemans/Bayer multistix 8SG) provided by the study and record the urine sampling method and dipstick result on the CRF. The urine sample will then be split, if sufficient quantity is available, with the priority fraction being sent to the local NHS laboratory for routine diagnostic processing, and the second ‘research’ fraction being sent to the SACU(Specialist Antimicrobial Chemotherapy Unit, Public Health Wales Microbiology Cardiff, University Hospital of Wales) reference laboratory: for more in-depth analysis. As only small volumes of urine (minimum 1 mL) are required for each laboratory, it is expected that for most urine samples, it will be possible to split the urine into the two fractions.

If a sample is not obtained during this visit the parent will be asked if they could obtain one at home, refrigerate it and return it to the primary care site within 24 hours. Where possible, the DUTY recruiter will offer to collect the urine sample from the patient’s home.

In a sub-sample of children recruited from a handful of sites linked to the Bristol study centre, we will use time-motion techniques to measure the additional time (parent and healthcare professional) taken to collect the urine sample and to perform dipstick testing during the primary care appointment. These data will be used to inform the economic analysis.

### Maximising urine samples

Obtaining the urine samples will be challenging, and a suboptimal return rate will diminish power and increase risk of bias. Therefore: (1) we will monitor the location of urine specimens using a web-based database. Clinical data for recruited children will be logged onto the secure study website. Dipstick urinalysis data may be added after the clinical data and will provide a record of the urine having been obtained. This will allow the research team to identify children for whom urine samples have not been provided, and to check with the relevant recruiter as to whether this should be followed up. Both the Research and NHS laboratories will also record the arrival of, and results from, the specimens on the website; and (2) each centre will provide dedicated DUTY recruiter resource to assist Option 1 practices with obtaining urines.

### Laboratory processing of urine samples

The NHS ‘clinical’ fraction will be labelled with the child’s unique DUTY study identification (ID) number on DUTY specific labels as provided in patient packs. Similar DUTY labels will be adhered to the DUTY study specific microbiology form and the sample sent to the local laboratories using the site’s normal method of transport. Any samples not collected within 4 hours will be refrigerated at the site and processed within 36 hours. Clinicians will receive and act on reports from their local laboratory as in the course of usual clinical care.

The remaining portion of urine will be decanted into a sterile monovette container containing boric acid. This will be labelled with the child’s study ID number and sent by 1^st^ Class Royal Mail using Post Office approved Safeboxes^TM^ to the central research laboratory.

### Minimising effects of sample contamination and assessment of asymptomatic bacteriuria

Contamination of urine (a cultured organism from a source other than the urinary tract) can lead to false positives: a potential false positive rate of 7.2% has been identified in one study by comparing pairs of urines from 203 children [32]. All nine (5.4%) children in this study with a mixed culture ≥10^5^ CFU/mL of uropathogens (a heavy mixed growth) in their first sample had a UTI excluded in the second [32]. In addition, bacteria at ≥10^6^ CFU/mL (have also been found in the urine of approximately 1.5% of young, asymptomatic, children when screened using the ‘gold standard method’, supra-pubic aspiration [33], and most did not experience long term sequelae [34]. Therefore, distinguishing UTI from asymptomatic bacteriuria and bacterial contamination is difficult, and could lead to spurious associations between symptoms (e.g. diarrhoea) and apparent ‘UTI’ that is in reality contamination or potential harmLess asymptomatic carriage.

Clinicians use the presence of UTI symptoms to help interpret culture positive results but this leads to incorporation bias. In DUTY, we could restrict recruitment to those children with currently recognised symptoms of UTI, but since the purpose of DUTY is to determine the strength of association between currently recognised as well as *currently unrecognised* symptoms/signs and UTI, it is important that eligibility criteria are as ‘open’ as possible (and that a prospective cohort, as opposed to retrospective case–control, design is used), but without including children in whom a positive culture is unlikely to be clinically relevant (e.g. a well child with conjunctivitis). Therefore, DUTY will recruit children with constitutional and/or urinary symptoms and make the assumption that the presence of no more than two pathological bacteria of at least >10^3^, but usually >10^5^ CFU/mL on culture of their urine is clinically significant. This could result in more urine samples being tested and more children receiving antibiotics than is strictly necessary, but carries the benefit that more UTIs would be identified and treated promptly.

### Data collection

Unique study identification numbers will be sequentially generated and used on pre-printed consent forms, paper CRFs, urine sample labels and test request forms (for local NHS and central research laboratories).

A CRF will be completed for all consented patients. This will include a short medical history including recent antibiotic use and other potential risk factors for UTIs and resistance, and clinical examination findings. An outline of the domains covered in the CRF can be found below in the next section.

### Case report form

The CRF will contain as many of the known and potential features associated with UTI as are feasible without overly compromising the speed and simplicity of completion.

Five sections will facilitate data entry by different personnel (recruiter taking consent or responsible clinician) so as to minimise the burden to healthcare professionals undertaking same day primary care:

1. Eligibility screening and consent (to be completed by recruiting clinician within the recruitment interview with the parent).

2. Registration (to be completed by recruiting clinician as above): Socio-demographic data (to include: date of consultation, name, address, contact telephone number/s, ethnicity [21], date of birth and gender [9]). We will also ask about parent’s highest educational attainment level and their financial well-being in order to assign a measure of socio-economic deprivation.

3. Presenting Symptoms and Medical History (to be completed by recruiting clinician as above): child’s presenting symptoms will be recorded, along with known previous medical history (e.g. previous UTI, circumcision [5,35], child or family history of vesico-ureteric reflux [36], other abnormalities of the urinary tract, learning difficulties, details of prior surgery, other co-morbidities, recent and previous long-term use of medicines, including antibiotics).

4. Clinical Examination and Management (to be completed by child’s responsible clinician within a standard consultation): in addition to the ‘constitutional’ and ‘urinary’ study eligibility symptoms defined by NICE [9], we will collect information regarding the clinician’s global assessment of illness severity [37], respiratory and gastro-intestinal symptoms and signs, and the symptoms and signs proposed by NICE to distinguish ‘typical’ from ‘atypical’ UTI, such as poor urinary flow and abdominal mass.

Clinicians will be asked to record the child’s management, including antibiotic use and immediate referral to secondary care. To assess the diagnostic value of the urine dipstick test, we will ask clinicians to record their working diagnosis before having seen the dipstick results, and to record whether their working diagnosis has changed after they have seen them. Finally, for the economic analysis, we will ask clinicians to state what their management would be if the patient were not enrolled in the DUTY study (e.g. no urine test/not treated for UTI or urine test/treat for UTI). This will provide information on the ‘clinician judgement’ diagnostic strategy that will be a comparator in the economic evaluation.

5. Urine collection and processing (to be completed by recruiting clinician): Urine sampling method (clean catch or nappy pad) and urinalysis results with date, time of testing, with a prompt to inform the responsible clinician of dipstick result and confirmation that the sample has been sent to the local NHS and central research laboratories.

In addition to the CRF, the study web-based data collection platform will include additional sections to cover data entry for the following: (1) NHS microbiology laboratory microscopy and culture; (2) SACU research laboratory microscopy and culture; (3) patient follow-up at Day 14 following recruitment, and (4) patient notes review at 3 months from recruitment. Further detail of the content of these domains is provided below.

### Processing of urine samples by NHS laboratories

NHS laboratories will be informed of the study and the agreement of the lead consultant microbiologist, laboratory manager and the NHS hospital Research and Development approval obtained before patient recruitment begins. The laboratories will be asked to complete the following tasks for DUTY study samples:

1. Log the date and time of specimen arrival on the secure DUTY web-based database.

2. Process the urine and report the result back to the requestor using their own Standard Operating Procedures (SOPs) and Laboratory Information Management Systems (LIMS).

3. Enter the results of urine culture onto the DUTY study web-based database. Since laboratories vary in their SOPs, not all of the following will be available however, microscopy for white and red cells; quantification and purity of bacterial growth; and speciation will be requested. Laboratories will be asked to transcribe this information onto the DUTY web-based database in order to activate laboratory payment.

4. Store any isolates from urines with >10^5^ CFU/mL in pure/predominant ureopathogen growth for referral onto the central research laboratory at the end of the study. These should be stored, ideally on cryogenic beads, at temperatures of -70 C, or on slopes at -20 C if -70 C storage facilities are not available.

### Processing of urine samples by the research laboratory

The central research laboratory has experience in supporting other primary care UTI studies and performed a similar role to that described below in the previous EURICA study (Epidemiology of urinary tract infection in children with acute illness in primary care) [3].

1. Urines will be sent overnight by Royal Mail SafeBoxes^TM^ by the participating sites. Boric acid will be used to stabilise bacterial counts.

2. On receipt at the central research laboratory, the urine sample will be spiral-plated on blood agar and UTI Chromogenic agar will be used to quantify bacteria >2x10^1^ CFU/mL and <10^10^ CFU/mL.

3. The bacteria will be identified to species level and stored on cryogenic beads at −80°C. The urine will be stored frozen.

4. Results will be recorded on a designated laboratory worksheet and entered into the DUTY web-based database.

Where the urine culture result produced by the central research laboratory is positive and the local NHS laboratory result is negative or not processed, and if this discrepancy is considered by the lead SACU microbiologist to be clinically significant, we will inform the child’s responsible clinician. This will enable the child’s responsible clinician to consider the future management of the child in the light of the additional information arising from the study.

### Patient follow-Up

#### Telephone follow-up at Day 14

Each centre will telephone parents of all children selected for follow-up according to the proportional selection rules in Table 2, to record symptom duration and healthcare resource use (e.g. repeat primary care contacts other community care, secondary care contacts, prescribed and over-the-counter medications) during the 14 day period after recruitment. Parents will also be asked to detail expenses and time off work due to their child’s illness and rate the child’s quality of life (including symptoms, sleeping, feeding, behaviour and wellbeing) using a previously validated measure (TAPQoL) [38].

**Table 2:** proportional selection rules for DUTY follow-up

**Category**	**Definition**	**Location**	**Proportion to be sampled at Day 14**
> 10^5^ CFU/ml	Pure or 1 predominant species	BOTH NHS lab and Central research lab	100% (All)
>10^3^ and < 10^5^ CFU/ml	Pure or 1 predominant species	Central research lab	20% in total *(combination of both categories)*
>10^5^ CFU/ml	2 or more species	BOTH NHS lab and Central research lab	
< 10^3^ CFU/ml and ‘No Growth’		BOTH NHS lab and Central research lab	10%

### 3 Months note review

Each research centre will conduct a primary care notes review for all children who were selected for follow-up. Primary care contacts, medications and secondary care utilisation during the 3 months after study recruitment will be recorded during the review.

### Withdrawal & loss to follow-Up

In the majority of cases the only active participation of participants is at the initial consultation, and withdrawal from the study in most cases is unlikely. Attrition in those selected for 14 day follow-up due to the challenges of making contact with busy parents will be minimised by making several attempts to contact parents/guardians by telephone and, if this is unsuccessful, a postal version of the resource use questionnaire will be posted to participants with a stamped addressed envelope for return. Parents will be offered a £5 voucher, by post, on completion of follow-up either by telephone or post.

### Electronic data entry

The DUTY data collection process is complex and involves input from a number of different personnel at different sites: (1) CRF data by a combination of study DUTY recruiters, practice nurses, GPs and dedicated staff at recruiting primary care sites; (2) clinical and research urine culture results by laboratory technicians and managers; and (3) follow-up data at Day 14 and 3 months by study centre administrators and research nurses. To optimise the quality of the data entry and to enable effective data collection from multiple sites across England and Wales, we decided to use a secure, web-based electronic data collection platform.

We will use a secure web 2.0 clinical study management system (The electronic Primary Care Research Network (ePCRN)). Hosted by South London and Maudsley (SLAM) NHS Foundation Trust, the ePCRN implementation is of a separate domain and a Citrix farm serving published applications, with a Structured Query Language (SQL) server providing clinical based study application databases. The system avoids potential data loss, duplication and security issues with laptops and portable media and has been approved by ethics and by the SLAM Caldicott and Executive Committees.

Web-forms for data collection will be created in ASP.net (a dynamic web application framework) on top of a dedicated SQL data management server, with data variables forced to comply with entry and validation rules defined in the data element definitions. The SQL data management server will incorporate auditing, backup and recovery facilities. The study workflow and algorithms will be enforced using the same methods, and a visual algorithm on the web pages will guide users. The web-based system will be piloted for ease of use prior to data entry go-live.

### Electronic data protection and confidentiality

The ePCRN safeguards the legal and ethic rights of service users through a fully integrated research security management system consisting of two component parts: (1) technical specifications built into the DUTY study database during the development phase, and (2) procedural standards governing the launch and day-to-day use of the application by DUTY study researchers.

Access to users will be provided through study-specific logon points in Citrix Access Gateway Advanced Access Control. Citrix software establishes a secure, encrypted, connection with the user’s PC, allowing access from the Internet uniquely to the Citrix Access Gateway and enabling access to identifiable study data for authorized users.

### Data entry in primary care sites

In order to maximise the acceptability and ease of use of DUTY data collection tools, clinicians working in settings without web access or whose working practice made web data entry an unwelcome burden, will be able to opt for paper-based data collection with the support of the local study centre in entering data, or making alternative arrangements for data entry, on their behalf.

The web-based data collection system will be presented as the preferred method of data collection, and practice-based recruiting staff will be strongly encouraged and supported to enter CRF data onto the database directly or, if using paper-based CRFs in the recruitment interviews, to retrospectively enter the data in a timely way (consent and registration within 24 hours, and full eCRF data within 5 working days).

### Data entry in the local NHS laboratory and central research laboratory

Once in the local NHS and SACU research laboratories, staff will be able to access an anonymised data collection page, where only study numbers and the data collection forms for the urine samples can be seen. Laboratory staff will be asked to log the samples on receipt and enter the results when available.

### Follow-up data entry in research centres

At day 14 from recruitment, and at 3 months, research staff will enter symptom duration, healthcare resource utilisation and expenses data from telephone interviews and practice records respectively onto web-based data collection forms.

### Analysis

The overall aim of the analyses is to derive a validated clinical prediction rule for UTI among acutely ill children presenting to primary care.

### Sample size calculation

To estimate the required sample size we drew on our experience with the EURICA study, which found a UTI prevalence rate in children aged before their fifth birthday of 4% [3]. We considered first the strength of association between candidate predictors (symptoms, signs or dipstick results) and UTI as well as the precision of the final algorithm’s sensitivity for the detection of UTI. Taking the most conservative assumptions, i.e. candidate predictors present in 10% of children and an overall UTI prevalence of 2%, 3,000 urine sample results are required to detect an odds ratio of 2.4 with 80% power and a two-sided alpha of 5%. With an overall prevalence of UTI of 2%, an algorithm sensitivity of 80% and 3,100 urines, the 95% confidence interval (CI) will be no more than +/−10%. We propose to recruit 4,000 children with a target of recovering urines from at least 77.5% for algorithm derivation and a further 2,000 children for validation.

Given the complexity of the statistical analyses, large number of possible predictors and the need to account for some missing data in predictor variables, we propose to recruit at least 7000 children in total (two-thirds for algorithm derivation; one-third for validation), in order to maximise the statistical power of the sample).

### Statistical analysis

#### Defining the primary outcome

The first stage of the analysis will investigate the best combination of microbiology data from the local (NHS) laboratories and the central research laboratory that can be used to define urine samples as positive for UTI. We will agree definitions of UTI positivity for data from each laboratory (using culture results and white cell counts) and will cross-tabulate these. Possible disagreements will be examined, and samples classified after discussion as: (1) “Agree UTI negative”; (2) “Agree UTI positive”; (3) “Disagree (CL positive, LL negative)”; (4) “Disagree (CL negative, LL positive)”. If overall between-laboratory reliability (classified on kappa statistics) is good, the primary outcome will be defined as positive for samples classified as (2) “Agree UTI positive”. If overall agreement is moderate or poor, we will explore reasons for this. We will stratify according to age of the child and method of collection (clean catch or nappy pad), and investigate whether reliability varies between strata. We will also examine the inference of laboratory methods (e.g. whether samples are process in boric acid) and time from sample collection to laboratory processing.

If the best definition of UTI positivity remains unclear, then we will select a small number of signs and symptoms that the literature suggests are clearly associated with presence of a UTI. We will examine associations of UTI positivity with these symptoms, using different definitions of positivity (e.g. based on central or local laboratory, threshold for amount of growth, evidence of growth of other species, method of sample collection. We will select the best definition of microbiological positivity based on the magnitude of associations with the selected signs and symptoms.

### Descriptive analyses

We will use methods appropriate for small proportions [39] to estimate the prevalence (with 95% confidence interval) of culture positive urines in acutely unwell children aged before their fifth birthday presenting in primary care. This will be undertaken on the whole dataset. The degree of variation in prevalence between practices and geographical areas will be explored using two level random-effects logistic regression models (with practice/site as a random effect and area as a fixed effect). This analysis will also explore difference by recruitment site type (general practice, WICs and CEDs). Children in whom urine samples are obtained will be compared to those who are recruited, but no urine sample is obtained in terms of clinical presentation and demographics.

We will compare the probability of contamination in samples that are retrieved via a ‘clean catch’ method with those using nappy pads, controlling for patient and practice factors in a two level random-effects logistic regression model (objective 4). We will examine the impact of timing of sample in relation to the time between obtaining the urine transportation (including day of the week) and laboratory analysis on the rates of positive and contaminated urine samples (e.g. exploring if delayed samples such as those taken after daily laboratory collection have an impact on contamination rates).

The sample will then be sub-divided into algorithm derivation and validation datasets, compromising two-thirds to one-third of the data set, respectively. This will be done by randomly selecting practices: all of their patients will then contribute to one of the two datasets.

### Development of clinical prediction rules

We will develop a clinical prediction rule based on the linear predictor in a logistic regression model in which the outcome variable is a culture-positive urine result. Candidate diagnostic variables will be categorised into demographic background and medical history (for example, gender, previous UTI); both specific and general systemic presenting symptoms and signs (for example overall illness severity, fever, vomiting); and results from urine dipstick analysis (nitrite, leukocyte esterase, protein, blood and glucose). Because of concerns that some aspects of medical history or demographics may be associated with asymptomatic carriage rather than active infection, we will also develop a ‘signs and symptoms’ only prediction rule. Variables will be included in logistic regression models based on an “inclusive” p value threshold of 0.1. We will check for nonlinear effects of continuous variables, and will examine candidate interactions specified *a priori*. Any further candidate interactions will be agreed before analyses commence. Such effects will be included in the final models as necessary.

We will begin by examining the predictive value (based on diagnostic odds ratios and C statistics) of the best predictors from each of the three categories (socio-demographic and previous medical history, clinical assessment, and dipstick urinalysis) of variables. We will then examine the additional diagnostic value of presenting signs and symptoms (compared with socio-demographic and medical history alone) and of dipstick results (compared with the other two categories). We will examine whether it is possible to identify subgroups of children in whom dipstick testing is and is not justified based on their signs and symptoms. The final algorithm will be characterised based on its sensitivity and specificity, and positive and negative likelihood ratios.

### Validation of clinical prediction rule

Diagnostic models that are developed using *p*-value-based variable selection will inevitably suffer from statistical over-optimism. Therefore, the final models will be validated using the second dataset, and the published rule will be based on the linear predictors from the model re-estimated in this validation dataset. A comparison will be made between the results obtained from the validation and the use of shrinkage based approaches applied to the original development dataset [40]. A comparison will be made between the results obtained from the validation dataset and the use of shrinkage based approaches applied to the original development dataset. The magnitudes of regression coefficients, and overall diagnostic value of the linear predictor, will be compared between the primary outcome and other definitions of culture positivity.

### Analyses of follow up data

Children with positive urine cultures (‘contaminants’ and ‘UTIs’) who the clinician felt at recruitment had a suspected UTI will be compared to those who the clinician felt there was little probability of a UTI in terms of their subsequent illness course and resource usage over the next three months.

### Risk stratification for clinical practice

In the final stages of analysis, we will examine the sensitivity and specificity of the linear predictor, based on a set of chosen thresholds for positivity. This will be used to identify several candidate clinical prediction rules for comparison in the economic evaluation. We will select thresholds that provide a range of clinical prediction rules from high sensitivity/low specificity to low sensitivity/high specificity.

### Minimising Bias

The following design and analytic strategies will be employed to minimise bias:

(1) Selection bias: where possible we will recruit consecutive children. We will ask sites to keep a screening log of patients approached but who did not take part in the study and reasons for this;

(2) Index test technology: all tests (symptoms, signs, nappy pads, dipstick tests) will be carried out using standardised equipment and protocols;

(3) Incorporation bias: the reference standard will consist of culture alone and will not incorporate any of the index tests;

(4) Review bias: observers assessing the index tests will differ from and be blind to those assessing the reference standard (and vice versa);

(5) Verification bias: all children who contribute to the study will have a urine sample sent to assess the reference standard. Children in whom it is not possible to obtain a sample will be excluded from the analysis. It is unlikely that reasons for failure to obtain urine samples will be related to the index tests but we will compare children with and without urine cultures;

(6) Disease progression bias: we expect the time between clinical assessment and obtaining the urine samples to be minimal (no more than 24 hours);

(7) Treatment paradox: for most children, antibiotic treatment will be started after the urine sample has been obtained, but we will record where this has not been possible;

(8) Handling of indeterminate or uninterpretable results or withdrawals: these parameters will be measured and considered in the analysis, and;

(9) Appropriateness of the reference standard: use of >10^3^, but usually >10^5^ CFU/mL of one, but no more than two uropathogens is likely to detect the majority of children with UTI, but the second ‘research’ urine result from the SACU laboratory will allow for sensitivity analyses around different bacterial concentrations. Where possible, we will measure all threats to validity (e.g. time between clinical assessment and obtaining and culturing the urine sample) that could influence results.

### Economic analysis

The aim of the economic analysis is to compare candidate clinical prediction rules (CPRs) on: (a) incremental cost per correct diagnosis of bacteruria, b) incremental cost per symptomatic day avoided, and c) incremental lifetime cost per quality adjusted life year, including potential long term complications of UTI from NHS and societal perspectives.

The cost-effectiveness of each candidate prediction rule will be compared to a ‘clinical judgement’ testing strategy and two ‘boundary strategies’: 1) Performing a urine test in every child meeting the DUTY eligibility criteria, and 2) No testing, the diagnosis of UTI is made clinically. The two boundary strategies are not intended to reflect clinical reality, but provide a reference point against which other diagnostic strategies can be compared. The cost-effectiveness of each candidate CPR will be assessed against these three strategies using a decision analytical model. The face validity of the model structure will be reviewed by clinicians on the DUTY study team before being finalised.

### Cost per correct diagnosis of UTI (diagnostic model)

The diagnostic cost-effectiveness model requires information on cost (cost of sample collection, cost of NHS laboratory testing), probabilities (probability of sample being obtained and probability of sample being contaminated) and diagnostic accuracy parameters (sensitivity and specificity of the various diagnostic strategies). The diagnostic accuracy parameters will be derived directly from the DUTY CRF and the statistical analysis described above. The probability of sample collection and contamination will be observed in the DUTY study. The cost of sample collection and laboratory testing will be derived from a combination of surveys of NHS laboratories and GPs and a time motion study observing primary care clinical staff as they collect and process urine samples. The model will estimate the cost per patient correctly diagnosed. The model will also include diagnostic strategies that incorporate dipstick testing (e.g. in those children thought to be a moderate risk of bacteriuria) with information on a small number of additional parameters (i.e. cost, sensitivity and specificity of dipstick testing).

### Cost per symptomatic day avoided (short term model)

The model will then be extended to estimate cost per symptomatic day avoided at day 14. A Markov model will estimate the short-run cost-effectiveness of each diagnostic strategy. This extension will require information on additional parameters including the probabilities of receiving antibiotics and hospital admission in children diagnosed with and without UTI, the cost of antibiotics and hospital admission/testing, and the daily recovery probability for children with bacteriuria treated with antibiotics, children with bacteriuria not treated with antibiotics and children without bacteriuria. The probabilities of antibiotic treatment and hospital admission will be based on 14 day interviews and review of patients’ primary care medical notes at 3 months. The daily recovery probabilities will be based on 14 day interviews with parents of children selected for follow-up. As all children in DUTY will receive a urine test, we will not be able to observe the recovery of children with bacteriuria not treated with antibiotics (i.e. false negatives). For this transition probability we will use literature estimates of the effect of antibiotic therapy on symptom duration.

### Cost per quality adjusted life year (QALY -long term model)

The structure of the long-run Markov model will be based on the a previously developed model [27]. The model will provide a link between the number of UTI attacks that a child will experience, the proportion that are pyelonephritic, the prevalence of vesicoureteral reflux, the probability of progressive renal scarring, the risk of end stage renal disease (ESRD) and disease management. Outcome parameters such as probability of UTI recurrence, renal scarring, ESRD, survival and utility values of renal disease will be based estimates for the sensitivity and specificity of imaging tests for VUR (e.g. ultrasound, MCUG), costs of treatment (e.g. antibiotic prophylaxis, cost of pyelonephritic treatment, dialysis, transplant), recurrence of UTI, disutilities (pyelonephritic, dialysis, transplant) and survival [27].

All analyses will be probabilistic, as all parameters will be entered into the model as distributions. Therefore the results will be presented as cost-effectiveness acceptability curves (CEACs). The CEAC demonstrates which of several testing strategies is most likely to be cost-effective at any fixed willingness to pay for a correct diagnosis. Costs and outcomes occurring after the first year will be discounted at 3.5%. We will use net monetary benefits and cost-effectiveness acceptability curves, at plausible willingness to pay thresholds (e.g. £0 to £50,000 per QALY) to identify the most cost-effective diagnostic strategies. Deterministic sensitivity analyses will also be used to evaluate the impact of key parameters on results and the influence of various CFU/mL thresholds on the choice of clinical prediction rule.

## Discussion

This paper describes a diagnostic and prospective observational study in primary care, that aims to recruit at least 7,000 children aged before their fifth birthday, who are assessed for any acute, non traumatic, illness of ≤ 28 days duration. Urine samples will be obtained from eligible consented children and tested with a dipstick before being sent to a local NHS laboratory and a central research laboratory for microbiological analysis.

This study will provide novel, clinically important information on the diagnostic features of childhood UTI to help primary care clinicians improve their diagnostic efficiency. New insight into the diagnostic value of dipstick urinalysis and urine sampling methods will also be provided.

The observational design of the study will minimise disruption to normal practice and reduce the research burden on healthcare professionals, thereby maximising the applicability and generalisability of findings. In addition, this study will increase awareness of UTI as a possible diagnosis in appropriate children under 5 years old. We will collect full clinical information, including presenting symptoms and signs, medical history and clinical diagnosis; urine dipstick data and culture data from both usual routine local NHS laboratories and central research laboratory. All the above will be utilised for derivation and validation of the diagnostic algorithm. In addition the follow-up data at 14 days and 3 months will allow for full health economic analysis, providing cost effectiveness outcomes. Additional useful information such as the best way of sampling urine from young children in primary care, the species and sensitivities of the infecting organisms, and contamination rates will also inform care in the future.

The main challenge to the DUTY study is the large number of participants and urine samples needed. A substantial number of practices across each of the four centres will need to be recruited, to provide a large enough population of potential participants. In addition to this, parental consent will be required at the same general practice visit when they are invited to participate, meaning there is a reliance on parents being willing and having the time to participate immediately. Despite the dedicated DUTY recruiting staff on the ground, who will provide support to practices, study buy-in is needed from all primary care staff to ensure adequate resources and infrastructure are in place to facilitate the conduct of the study. This will require training of all staff involved at each participating primary care site. A major contribution from NHS microbiology laboratories will also be necessary, for processing the increased number of urine samples received from primary care sites.

This study will rely on sampling methods most commonly used currently in UK primary care, namely nappy pad and clean catch. Suprapubic aspiration and catheterisation methods are neither feasible nor appropriate for primary care. Nappy pad sampling may carry an increased risk of contamination or un-interpretable results. This may lead to the exclusion of some results from the main analysis. However, this will be minimised by obtaining clean catch samples where possible. Lastly, the electronic data capture on the database has been designed to allow for all parties to enter data separately. A challenge will be to ensure all data is entered into the database in a timely way, to allow for both the real time monitoring of recruitment and urine sample location, as well as the conduction of the follow-up interviews within the tight timeframe.

In summary, this will be one of the largest studies of its kind undertaken in primary care, involving obtaining clinical samples from children, and will help guide management of the acutely unwell child, which is a common and important aspect of primary health care delivery. Improved assessment and diagnosis may lead to more appropriate microbiological and point of care testing and more timely treatment and investigation of those children who are most likely to benefit, while reducing unnecessary treatment and investigation among those that are most unlikely to benefit. This is likely to improve outcomes for individual patients and may help prevent long-term sequelae. The overall outcome of the study will be to achieve a more consistent approach in the clinical care of a common condition, based on accurate diagnosis and effective clinical management.

## Endnotes

^a^ This paper will use the term ‘parent’ to refer to the person with legal responsibility for the child, therefore as applied in this paper the term also encompasses carers (foster parents, legal guardians etc.).

## Competing interests

The authors declare that they have no competing interests.

## Authors’ contributions

CCB and ADH are co-chief investigators and act as guarantors of the study in its entirety. CCB and ADH jointly led the development of the research question, study design and implementation of the study protocol, along with KH, JS, WH, PL, BD, RH, MW and AM. HD is Study Manager and coordinated the operational delivery of the study protocol across the UK. ETJ and CAW are study managers at Cardiff, and coordinated the delivery for the study protocol in Wales. PL and BD are principal investigators, responsible for study oversight at Southampton and London, respectively. RH, MW and AM have provided expert microbiology input. HD, ETJ, MG and CAW jointly drafted the manuscript. All authors listed provided critical review and final approval of the manuscript.

## Pre-publication history

The pre-publication history for this paper can be accessed here:

http://www.biomedcentral.com/1471-2334/12/158/prepub
